# Effect of an hyperbaric nitrogen narcotic ambience on arginine and citrulline levels, the precursor and co-product of nitric oxide, in rat striatum

**DOI:** 10.1186/2045-9912-1-16

**Published:** 2011-07-05

**Authors:** Nicolas Vallée, Jean-Jacques Rissoe, Jean-Eric Blatteau

**Affiliations:** 1 Institut de Recherches Biomédicales des Armées-Antenne Toulon. Equipe Résidente de Recherche Opérationnelle, BP 20548, 83041 Toulon Cedex 9, France

## Abstract

Previous studies performed in the laboratory have shown that nitrogen narcosis induces a decrease in striatal glutamate and dopamine levels. Although we stimulated the N-methyl-D-aspartate (NMDA) receptor, an important glutamate receptor required for motor and locomotor activity managed by the striatum, and demonstrated that the receptor was effective when exposed to nitrogen at 3MPa, it was not possible to return the striatal glutamate level to its base values. We conclude that it was the striatopetal neurons of the glutamatergic pathways that were mainly affected in this hyperbaric syndrome, without understanding the principal reasons. Hence we sought to establish what happens in the vicinity of the plasma membrane, downstream the NMDA-Receptor, and we used the hypothesis that there could be neuronal nitric oxide synthase (nNOS) disturbances. A microdialysis study was performed in rat striatum in order to analyse levels of citrulline, the NO co-product, and arginine, the NO precursor. Those both NO metabolites were detectable with an HPLC coupled to a fluorimetric detector. Exposure to pressurized nitrogen induced a reduction in citrulline (-18.9%) and arginine (-10.4%) levels. Under the control normobaric conditions, the striatal NMDA infusion enhanced the citrulline level (+85.6%), whereas under 3 MPa of nitrogen, the same NMDA infusion did not change the citrulline level which remains equivalent to that of the baseline. The level of arginine increased (+45.7%) under normobaric conditions but a decrease occurred in pressurized nitrogen (-51.6%). Retrodialysis with Saclofen and KCl in the prefrontal cortex under normobaric conditions led to an increase in striatal levels of citrulline (+30.5%) and a decrease in arginine levels (-67.4%). There was no significant difference when nitrogen at 3MPa was added. To conclude, the synthesis of citrulline/NO is reduced in nitrogen narcosis while it seems possible to activate it artificially by infusion. We have suggested that the low glutamate levels recorded in nitrogen narcosis induced these dopamine and NO reductions in the striatum.

## Introduction

**Figure 1:**
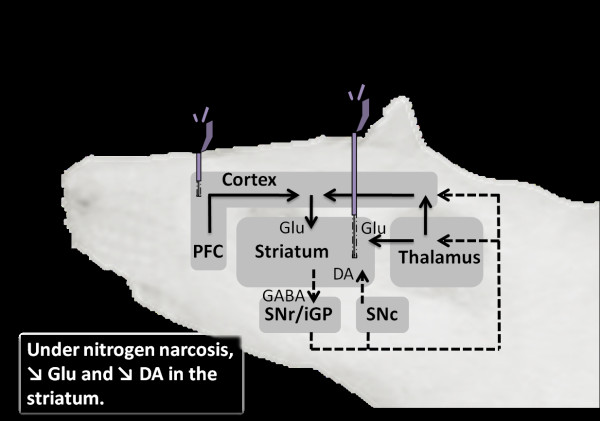
**Stimulation of the main striatal projections involved in nitrogen narcosis**. Two microdialysis probes are presented on the diagram to point out the stimulation points on the glutamatergic ways. The aim is to enhance neurotransmitters release and their metabolites in the striatum. A probe is schematized in the striatum and the second one is in the prefrontal cortex. Measures of citrulline arginine dopamine and glutamate are performed in the striatum. SNr/iGP projects to thalamus with GABA-ergic neurons. Thalamus essentially projects to striatum and cortex with glutamatergic neurons. The compilation of these both pathways virtually results in an inhibitory pathway, represented on the diagram. Cortex and especially prefrontal cortex (PFC) essentially project to striatum with glutamate as (excitatory) transmitter. SNc projects to striatum using dopamine, but the effect on striatum (inhibitory or excitatory) depends on the type of receptor: D1-like (direct pathway; inhibitory) or D2-like (indirect pathway not shown: excitatory). Using the direct pathway, striatum project to SNr/iGP with GABA. DA, dopamine; GABA, gamma aminobutyric acid; Glu, glutamate;PFC, prefrontal cortex; SNc, subtantia nigra pars compacta; SNr/iGP, substantia nigra pars reticulata and internal globus pallidus. Dotted arrows mark inhibitory pathways. Solid arrows mark excitatory pathways.

Considering motor and locomotor dysfunctions occurring under the effect of nitrogen narcosis [1], also known as diver's staggers, previous studies conducted at the end of the 1990s logically focused on the dopaminergic transmission in the rat striatum. A decrease in striatal dopamine levels was highlighted [1,2]. And yet, the control of motor and locomotor activity by the striatum requires dopaminergic as much as glutamate receptors. Hence, the logical follow-on from the earlier work was to extend the research to this other main neurotransmitter-glutamate-and its regulatory pathways, particularly as Abraini [3] had early suggested that inert gases under pressure may act on protein receptors such as the NMDA (N-methyl D-Aspartate) Receptor, a glutamate receptor. Hence our investigation described a decrease in striatal glutamate levels in rats subjected to nitrogen under pressure [4]. The logical consequence was to test proximal and distal stimulation of the striatal glutamate release (Figure 1), but no improvement was observed in relation to rat behavioural disorders. In fact, neither the intrastriatal NMDA infusion nor the KCl-mPFC (motor Prefrontal Cortex area) stimulation was effective in rectifying the nitrogen-induced glutamate reduction in the striatum [5,6]. Nevertheless, we demonstrated that the NMDA-receptor remains functional in the striatum, like in the substantia nigra [7,8], under the effect of nitrogen narcosis, as it increases extracellular dopamine levels. Even so, the increased effect on glutamate release noted under atmospheric pressure disappeared under pressurized nitrogen. We suspected a disturbance close to the plasma membrane on the intracellular side, behind the NMDA-receptor and we decided to focus on Nitric Oxide (NO). In fact, nitrogen under pressure has even been suggested by Vjotosh *et al. *in 1999 [9] to affect nitric oxide (NO) synthesis. We are the first to describe NO development under nitrogen narcosis.

Actually, the retrograde action of endogenous NO is known to regulate the synaptic level of various neurotransmitters [8-15] by modulating endocytosis, vesicle maturation [14] and the opening frequency of the NMDA-receptor [16]. For instance, a molecular analysis had revealed a direct action site for NO on the NMDA-receptor [16]. Besides, the neuronal NO Synthase (nNOS) produces NO almost exclusively after activation of the NMDA receptor [17-19]. The gaseous neuronal messenger in turn modulates glutamate transmission [20-24], but this is not all. NO diffusion is also known to modulate DA release in the striatum [15,25,26].

NO is synthesized from a unique precursor, arginine [27,28], by NO synthase (NOS) (see Garthwaite & Boulton [25] for review). NOS produces as much NO from arginine as it does the by-product, citrulline. The entire citrulline-NO cycle [29], which consists of an enzymatic recycle of arginine from citrulline, is present in the striatum [30-33].

The aim of this study was to check whether neuronally derived NO levels were changed under nitrogen narcosis, and what was the influence of this change on extracellular striatal glutamate and dopamine concentration. Arginine and citrulline concentrations were measured by microdialysis, and compared with glutamate and dopamine developments whether the striatum was stimulated or not. Our hypothesis was that a reduction in striatal glutamate and dopamine levels recorded under the effect of nitrogen narcosis could be coupled with a decrease in arginine and citrulline levels, the precursor and the co-product of NO. These metabolites measurable by a fluorimetric HPLC should reflect the NO synthesis. Complementary results of this study, essentially concerning dopamine and glutamate levels, have been presented in previous papers [4-6].

## Materials and methods

### Animals and ethical approval

All procedures for the use of animals were in accordance with the Council of the European Union rules (Brussels, Belgium), Directive of November 24, 1986 (86/609/EEC), as stated in French law (Decree 87/848), and experiments were conducted in accordance with the policies of our Institutional Animal Care and Use Committee (associated with Agreement Number: B13.0005.8). Male Sprague-Dawley rats (Charles River, France) weighing 300-350 g were used (n = 46). The rats were kept at 22 ± 1 °C in a 12-hour light/12-hour dark cycle (lights on at 7:00 a.m.) with food (A03, UAR) and water available ad libitum. After surgery, the rats were housed individually in Plexiglass^® ^cages where they recovered for at least a week before undergoing the microdialysis procedure.

The animals were divided into 6 groups. The first group (_1_), using helium at 3MPa (n = 8), needs to be compared to the second one (_2_) developed under nitrogen at 3MPa (n = 6). The aim was to dissociate the narcotic potency of nitrogen from the effect of the pressure per se. At this pressure, the narcotic potency of helium is very low. Hence, at the same pressure, we should only measure the narcotic potency of nitrogen. Two other groups aimed to activate the intrastriatal glutamatergic pathway by stimulating NMDA-receptor by retrodialysis. One was developed as a control (_3_) and it was developed under atmospheric pressure (n = 8), while the other (_4_) was conducted under pressurized nitrogen (3MPa) (n = 6). The two last groups (_5_6_) were similar to previous experiment (under atmospheric and hyperbaric condition; n = 9/9) except that the stimulation of the striatum was conducted from the prefrontal cortex using a KCl solution.

### Surgery

Anaesthesia was induced by halothane (5% with O_2_) (Halothane^®^, Belamont) and then deep anaesthesia was prolonged with sodium pentobarbital (30 mg/kg, i.p.) (Sanofi Santé Animal) and ketamine (0.40 mg/kg, i.m.; Imalgène^® ^500, Laboratoire Rhône-Mérieux). The rats underwent stereotaxic implantation with intracerebral guides (CMA/12 guide cannulae; Phymep, France): the coordinates of the striatum (group 1 to 6) were inter-aural; anterior 10.0 mm, lateral ± 2.8 mm, and height 6.4 mm; and those (group 5 and 6) for the prefrontal cortex were inter-aural; anterior 13.0 mm, lateral ± 1.4 mm, and height 8.4 mm, according to the Paxinos and Watson brain atlas [34]. The disposition and number of probes implanted depend on the stimulation.

### Microdialysis in hyperbaric conditions

The microdialysis equipment consisted of micro-injector pumps (CMA/102; Phymep, France) customized to support high pressure, a Raturn™ cage (Bioanalytical Systems, Inc.) to prevent fluid lines tangling, and probes (CMA/12; Phymep, France). Probes with a 3 mm long membrane were used for striatal microdialysis, and probes with a 1 mm long membrane were used for retrodialysis in the prefrontal cortex. HPLC switch valves, equipped with a 20 μl loop, were added to the microdialysis system, in order to propel the dialysate into the refrigerated microfraction collector (Univentor 820 Microsampler; Phymep, France) placed outside the hyperbaric chamber. The various components of the microdialysis devices were interconnected using FEP-tubing (1.2 μL/10 cm) or PEEK-tubing (1.3 μL/10 cm), and tubing adapters. The system was infused with artificial cerebrospinal fluid (CSF in mmol/L: NaCl 147, KCl 2.7, CaCl_2 _1.2, MgCl_2 _0.85). This system was designed to collect regular samples in order to freeze them as soon as possible, with no limitation on the number of vials available in the collector if the latter was placed inside the hyperbaric chamber [4].

The day before the experiment, new microdialysis probes were rinsed for 20 minutes with 70% ethanol in order to wash out the glycerol used for packaging. Each probe was then connected to the inlet microdialysis line for the hyperbaric chamber and checked for air bubbles. Then, when connected to the outlet line, the entire dialysis line was washed with CSF overnight. Between experiments, the entire circuit was rinsed for at least one day, with methanol/water (40/60) and then air-dried.

**Figure 2:**
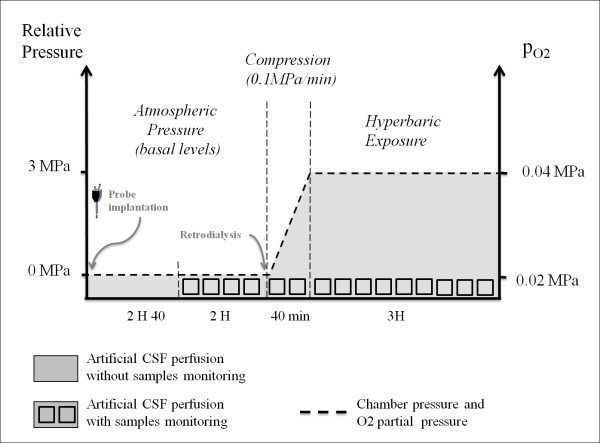
**Diving procedure**. Following the insertion of the probes into the brains, the rats were allowed a recovery time of 2 hours and 40 minutes before the four blank microdialysates were collected to establish the baseline. Each square represents one dialysate sample collected every twenty minutes. The stimulation by retrodialysis and the compression stage (or simili under atmospheric pressure) began at the same time. Rats remained at the maximal compression (or simili) for 3 hours. The dotted line marks the pressure of the inert gas and the oxygen partial pressure.

On the day of the experiment (Figure 2), at about 7:30 a.m., microdialysis probes were inserted into the brains of the conscious rats. Extracellular striatal levels of amino acids and monoamines were allowed to reconstitute for 2 hours and 40 minutes before the blank microdialysates (1 hour 20 minutes → 4 samples) were considered as the basal value (baseline). Striatum dialysates were collected every twenty minutes at the rate of 1 μl per minute (20 μl/dialysate) and frozen.

### Stimulation

Control rats and rats subjected to nitrogen narcosis had an intracerebral guide implanted in the right striatum infused with CSF.

For the intra-striatum stimulation with NMDA [5], two syringes infused the two probes implanted in the right and left striatum and liquid switch connectors (CMA/110, Phymep France) made it possible to switch manually between the two perfusion lines, one of which contained the pharmacological agent for retrodialysis (NMDA 2 mM, Sigma, France).

For the distal stimulation of the striatal glutamate release [6], we stimulated the corticostriatal pathway and more precisely the prefrontal cortex (PFC) [35-37] with a depolarized concentration of potassium chloride (KCl) and in the presence of local GABAb receptor blockade-in order to have a long lasting effect of the KCl-as inspired by Harte & O'Connor [38] protocol. Two syringes infused the rat prefrontal cortex implanted with two probes and liquid switch connectors (CMA/110, Phymep France) made it possible to switch manually between the two perfusion lines, one of which contained the pharmacological agent (Saclofen 1 mM and KCl 100 mM, Sigma, France). Another probe implanted in the striatum for conventional dialysis was infused with CSF.

### Hyperbaric procedure

Striatum basal activities were monitored for 4 hours before compression was started. The experiments were conducted in a 150 L hyperbaric chamber fitted with three viewing portholes. Rats were subjected to compression (40 minutes → 2 samples) at a rate of 0.01 MPa/minute up to 0.1 MPa, and then at a rate of 0.1 MPa/minute up to 3 MPa. Monitoring was continued during the period spent under pressure (3 hours → 9 samples). Carbon dioxide levels were kept below 300 ppm by continuous circulation of gases in the chamber through a soda-lime canister. Oxygen partial pressure was adjusted to 0.04 MPa (diving quality oxygen, Air Liquide, France). A powerful fan ensured that oxygen was well mixed with the gases added to generate narcosis. Humidity (40-60%) was controlled with silica gel, and the ambient temperature was adjusted to 27°C [39], to prevent hypothermia and ensure that the rodents remained comfortable. Lights were on. At the end of the experiment, a lethal dose of nitrous oxide was injected into the hyperbaric chamber and the decompression was performed.

In the atmospheric experiments, rats were anaesthetized with halothane (5% with O_2_) (Halothane^®^, Belamont) and sacrificed by lethal injection of pentobarbital.

### Apparatus and chromatography

Citrulline and arginine analyses. The microdialysis striatal sample content was analysed by High Performance Liquid Chromatography (Shimadzu, DGU-20A3 Degasser, LC-20AB pump, SIL-10ADvp Autosampler, CBM-20A device controller) coupled with fluorimetric detection (Shimadzu, RF-10Axl Fluorescence Detector). HPLC was carried out with a reverse phase C18 column (3 μm, 200 × 3 mm, Phymep) stabilized at 25°C using gradient conditions, according the following schedule: at 1.0 mL/min; first 12 min, 15% of eluent B; the concentration of B was raised linearly to 28% in the next 20 min; then raised linearly to 70% in the next 35 min; and finally raised linearly to 80% at the same flow rate until the chromatography was completed (80 min). Eluent A consisted of a sodium acetate buffer (sodium acetate 0.01 M, 15% MeOH, triethylamine 2 mM, EDTA 0.3 mM, pH 9.3) and eluent B was similar to eluent A but with the alcohol content adjusted to 45% (sodium acetate 0.01 M, 45% MeOH, triethylamine 2 mM, EDTA 0.3 mM, pH 9.3). Measurement of amino-acid concentrations required pre-column derivatization (fixing of a fluorophore to the sample to produce fluorescent derivatives) with an ortho-phthaldialdehyde (OPA) reagent (OPA 37 mM, 2-mercaptoethanol 128 mM, 25% methanol, water, borax 24 mM, pH 10) mixed on line and added to the dialysate (v/v).

### Statistical Analysis

For each subject, the baseline was determined using the four samples taken prior to gas pressure exposure. Samples were next expressed as a percentage of the baseline, taken as the 100% value (baseline). Data for the whole group was noted using median and the 25 and 75 percentiles. Non-parametric statistical tests were used as the number of animals was small. Firstly, baseline groups were compared to the compression and exposure stages for each experiment type and each molecule, with a Kruskal-Wallis test followed by a Dunn test. The effects of striatum stimulation were then compared under exposure to nitrogen and in atmospheric conditions for each molecule development, and at each time point (Mann-Whitney test).

A differentiation was made between exposure during the period under compression (from 0.01 MPa to 3 MPa) and the period spent at maximum pressure (3 MPa).

## Results

In rat striatum, the medium concentrations of citrulline and arginine during the control period, were 1.36 ± 0.34 and 7.64 ± 1.82 μM respectively, without taking probe recovery into consideration.

### Extracellular citrulline levels

#### Effects of nitrogen narcosis in the striatum

**Figure 3:**
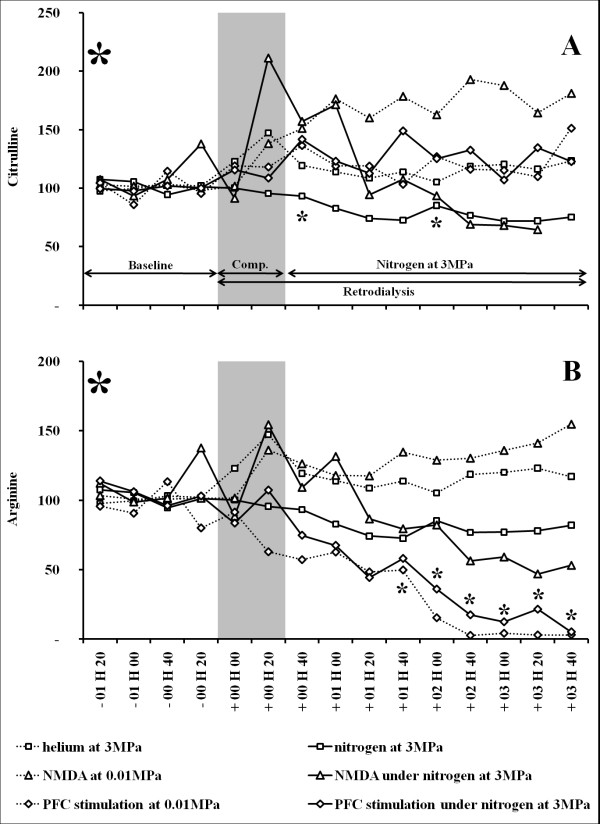
**(A-B) Effects of an NMDA stimulation or a KCL and Saclofen infusion under normobaric (n = 8/9) and high (n = 6/9) nitrogen-oxygen pressure atmospheres on levels of extracellular citrulline (A) and arginine (B) in the rat striatum**. A helium-oxygen (n = 8) mixture was used as the control in order to evaluate the effect of pressure *per se*, and it was compared to the nitrogen-oxygen (n = 6) mixture used in order to induce nitrogen narcosis (effect of narcosis and pressure are linked). The ordinate of each graph shows the median level of amino-acid expressed as the percentage of the baseline level, which is the mean of the four consecutive values observed immediately before the beginning of the compression/infusion of NMDA (2 mM) or KCl (100 mM) and Saclofen (1 mM). The time for compression (or simili) is shown by the grey area, which is followed by the maximum pressure period, 3 MPa (or simili). Dotted lines mark control experiments using helium at 3MPa or retrodialysis under atmospheric pressure. Lines mark experiments conducted in nitrogen at 3 MPa. Intergroup comparisons: strong signs in the corners of the graph indicate significant change in the evolution of citrulline or arginine levels (α = 0.05, Kruskal-Wallis test) between the nitrogen group and the NMDA-nitrogen group (#) or the KCl-Saclofen-nitrogen group (*). Small signs # and * at the corresponding time point indicate significant changes (α = 0.05, post hoc Dunn test) between the nitrogen group and the NMDA-nitrogen group (#) or the KCl-Saclofen-nitrogen group.

Exposure to pressurized nitrogen at 3 MPa (group 2) induced a reduction in extracellular citrulline levels, compared with the baseline (Table 1 and Figure 3A). The average decrease in citrulline reached 17.2% during the maximum exposure to pressurized nitrogen. When comparing with the basal values, the same experiment conducted under helium at 3 MPa (group 1) revealed significantly higher citrulline levels during the compression stage (up to 3MPa) but not during the stay at the maximal pressure. The maximal value reached 147.2%. Nonetheless, a significant difference (delta 20.4%) was noted when both experiments (group 1 vs. 2) were compared. (Table 1 and Figure 3A).

**Table 1:** Comparison of the striatal citrulline baseline with its evolution in nitrogen at 3 MPa (n = 6), in helium at 3MPa (n = 8), at atmospheric pressure with NMDA (2 mM) retrodialysis in the striatum (n = 8), or in nitrogen exposure with NMDA retrodialysis in the striatum (n = 6), at atmospheric pressure with KCL (100 mM) and Saclofen (1 mM) retrodialysis in the PFC (n = 9), or in nitrogen exposure with KCL and Saclofen retrodialysis in the PFC (n = 9).

Citrulline	Control groups		3MPa Nitrogen groups		Comparison	
	Comp.	**180 Minutes exposure Median (1**^**st **^**_ 3**^**rd **^**quartile)**	Comp.	**180 Minutes exposure Median (1**^**st **^**_ 3**^**rd **^**quartile)**	p-value	
	p-value	p-value post hoc test	p-value	p-value post hoc test	comp	Stay
**Helium at 3MPa **(1)**/Nitrogen **(2) n = 8/6	***0.002***	112.4 (91.3_151.7) 0.220	0.544	**82.8 **(67.5_97.7) ***0.005 ***	***0.008 ***	***< 0.001 ***
**NMDA retrodialysis **(3_4) n = 8/6	0.079	**168.5 **(142.7_216.3) ***< 0.001 ***	0.720	**94.3 **(58.7_169.1) 0.327	0.939	***< 0.001***
**PFC retrodialysis **(5_6) n = 9/9	***0.009 ***	**119.0 **(100.0_150.8) ***0.012 ***	0.141	**135.7 **(84.1_167.1) ***0.007 ***	0.397	0.727

-1- Baselines and periods at maximum pressure (but not the compression stage) were described using median and quartile. -2- The citrulline baseline was compared to the compression stage (up to 3MPa-"Comp.") and to a maximum pressure period (at 3MPa, "180 Minutes exposure") using a Kruskal-Wallis test (p-values were noted, α = 5%), followed by a post-hoc test transcribed by squares. Each square represents a string of samples (~median value) that must be compared with the baseline samples, arranged in chronological order: 2 squares for 2 series of samples available during the compression stage (or simili); 9 squares for 9 series of samples available during the period at the maximum pressure (or simili). Dark squares indicate a significant difference, as indicated by a post-hoc Dunn test (α = 5%), between samples at the corresponding time point and its baseline. White squares indicate no significant difference between the string of samples at the corresponding time point and the baseline. The compression stages (up to 3MPa) and the maximum pressure period (3MPa) of the control groups and the 3MPa nitrogen group were compared with each other, using a Kruskal-Wallis test (α = 5%). P-values were noted.

#### Effects of a proximal stimulation on the striatum

In atmospheric conditions (0.01 MPa; group 3), and compared with the baseline, NMDA infusion increased extracellular citrulline levels from the start to the end of the experiment. The increase in citrulline averaged 168.5%, compared with the baseline, and it reached 187.7%. When both conditions were combined, NMDA retrodialysis and high nitrogen pressure (group 4), extracellular citrulline levels remained unchanged from the compression stage to the end of the 3MPa period (Table 1 and Figure 3A). Citrulline levels were significantly higher when comparing values recorded during NMDA infusion in the atmospheric pressure group (group 3) with those recorded during nitrogen exposure with NMDA (group 4)(Table 1). Nonetheless, no significant difference was found between compression phases (Table 1). In comparison with the nitrogen exposure group (group 2), no significant difference in citrulline levels was shown with the nitrogen plus NMDA group (group 4) (during compression: n = 6/6, p = 0.571, α = 0.05; at 3MPa: n = 6/6, p = 0.225, α = 0.05).

#### Effects of a distal stimulation on the striatum

In atmospheric conditions (0.01 MPa) (group 5), and compared with the baseline, the PFC infusion increased extracellular citrulline levels from the start to the end of the stimulation (Table 1 and Figure 3A). Citrulline levels increased up to 119.0% compared with the baseline, and the maximum value (151.3%) was reached at the end of the experiment. In nitrogen at 3MPa with PFC retrodialysis (group 6), and compared with the baseline, extracellular citrulline levels increased from the compression stage to the end of the 3MPa period (Table 1 and Figure 3A). The increase in citrulline reached +33.7% during the period of maximum nitrogen pressure (Figure 2A). No significant difference was revealed when values recorded during nitrogen exposure (compression phase and stage at 3MPa) with PFC retrodialysis were compared with those recorded during PFC retrodialysis in the atmospheric pressure group (group 5 vs. 6) (Table 1). Data recorded in pressurized nitrogen at 3MPa (group 2) showed significantly lower citrulline levels than those recorded in nitrogen with the PFC stimulation (group 6) (n = 6/9; p < 0.001; α = 0.05); no significant difference is shown between the compression stages (n = 6/9; p = 0.183; α = 0.05).

### Extracellular arginine rates

#### Effects of nitrogen narcosis in the striatum

Exposure to pressurized nitrogen at 3 MPa (group 2) induced a reduction in extracellular arginine levels, compared with the baseline (Table 2 and Figure 3B). The medium decrease in arginine reached 6.3% during the maximum exposure to pressurized nitrogen pressure. The same experiment conducted in helium at 3 MPa (group 1) did not reveal significant changes in citrulline levels, except during the transient compression stage, which revealed an increase up to 147.2% (Table 2 and Figure 3B).

**Table 2:** Comparison of the striatal arginine baseline with its evolution in nitrogen at 3 MPa (n = 6), in helium at 3MPa (n = 8), at atmospheric pressure with NMDA (2 mM) retrodialysis in the striatum (n = 8), or in nitrogen exposure with NMDA retrodialysis in the striatum (n = 6), at atmospheric pressure with KCL (100 mM) and Saclofen (1 mM) retrodialysis in the PFC (n = 9), or in nitrogen exposure with KCL and Saclofen retrodialysis in the PFC (n = 9).

Arginine	Control groups		3MPa Nitrogen groups		Comparison	
	**Comp**.	**180 Minutes exposure Median (1**^**st **^**_ 3**^**rd **^**quartile)**	**Comp**.	**180 Minutes exposure Median (1**^**st **^**_ 3**^**rd **^**quartile)**	p-value	
	p-value	p-value post hoc test	p-value	p-value post hoc test	comp	Stay
**Helium at 3MPa **(1)**/Nitrogen **(2) n = 8/6	***0.031 ***	**100.8 **(84.0_115.2) 0.820	0.895	**93.7 **(83.1_98.4) ***0.031 ***	***0.008 ***	***< 0.001 ***
**NMDA retrodialysis **(3_4) n = 8/6	0.113	**131.8 **(103.6_192.2) ***< 0.001 ***	1.000	**71.7 **(46.3_121.1) ***0.013 ***	0.758	***< 0.001 ***
**PFC retrodialysis **(5_6) n = 9/9	0.141	**21.3 **(3.4_47.7) ***< 0.001 ***	0.426	**28.4 **(8.1_62.4) ***< 0.001 ***	0.709	0.188

-1- Baselines and periods at maximum pressure (but not the compression stage) were described using median and quartile. -2- The arginine baseline was compared with the compression stage (up to 3MPa-"Comp.") and to a maximum pressure period (at 3MPa-"180 minutes exposure") using a Kruskal-Wallis test (p-values were noted, α = 5%), followed by a post-hoc test transcribed by squares. Each square represents a string of samples (~median value) that must be compared with the baseline samples, arranged in chronological order: 2 squares for 2 series of samples available during the compression stage (or simili); 9 squares for 9 series of samples available during the stay at the maximum pressure (or simili). Dark squares indicate a significant difference, as indicated by a post-hoc Dunn test (α = 5%), between samples at the corresponding time point and its baseline. White squares indicate no significant difference between the string of samples at the corresponding time point and the baseline. The compression stages (up to 3MPa) and the maximum pressure period (3MPa) of the control groups and the 3MPa nitrogen group were compared with each other, using a Kruskal-Wallis test (α = 5%). P-values were noted.

#### Effects of a proximal stimulation on the striatum

In atmospheric conditions (0.01 MPa) (group 3), and compared with the baseline, NMDA infusion increased extracellular arginine levels, up to 145.7% on average compared with the baseline. The level of the arginine rapidly increases around 130% and it reached at the end of the stimulation 154.8%. When both conditions were combined (group 4), NMDA retrodialysis and high nitrogen pressure, a significant decrease was revealed in extracellular arginine levels finishing at 53.0% in the 3MPa period (Table 2 and Figure 3B). Arginine levels were significantly lower when values recorded during NMDA infusion in the atmospheric pressure group (group 3) were compared with those recorded during nitrogen exposure with NMDA (group 4) (Table 2). Nonetheless, no significant difference was found between compression phases (Table 2). The intergroup test (group 2 vs. 4) highlighted no significant difference in arginine levels when comparing the 3MPa nitrogen exposure group with the 3 MPa nitrogen exposure plus NMDA group (n = 6/6; p = 0.365; α = 0.05) (Figure 3A). No significant difference is shown between the compression stages (n = 6/6; p = 0.571; α = 0.05) (Figure 3B).

#### Effects of distal stimulation on the striatum

In atmospheric conditions (group 5) (0.01 MPa), as in nitrogen at 3MPa (group 6), and compared with the baseline, the PFC infusion decreased extracellular arginine levels (Table 2 and Figure 3B). Arginine levels decreased by up to 21.3% (and 28.4% in pressurized nitrogen) compared with the baseline, and minimum values (respectively 3.1% and 5.2%) were reached at the end of the experiment (Table 2 and Figure 3B). No significant difference was revealed when values recorded during nitrogen exposure with PFC retrodialysis were compared with those recorded during PFC retrodialysis in the atmospheric pressure group (group 5 vs. 6) (Table 2), whatever the phase. Nevertheless, values recorded in nitrogen at 3MPa combined with the PFC stimulation (group 6) were significantly lower than those recorded in nitrogen at 3MPa (group 2) (n = 6/9; p < 0.001; α = 0.05), the latter being lower than baseline values. No significant difference is shown between the compression stages (n = 6/9; p = 0.231; α = 0.05) (Figure 3B).

## Discussion

The main symptom of narcosis is major depletion in neurotransmission expressed by a significant reduction in striatal dopamine and glutamate levels (Table 3). We have now highlighted for the first time that extracellular citrulline and arginine levels were also reduced by nitrogen at 3MPa.

**Table 3:** Overwiew of the effect of nitrogen at 3MPa on rat striatal neurotransmitters, based on previous literature and the current study.

Levels of striatal ...	DA	Glu	Cit-NO
**Basal effect of nitrogen at 3MPa ...**	**down**	**down**	**down**
**... with NMDA retrodialysis**	**up**	**down**	**down**
compared to the basal effect	*more*	*same*	*same*
**... with PFC retrodialysis**	**down**	**down**	**up**
compared to the basal effect	*same*	*same*	*more*

Effects are reported after exposure to pressurized nitrogen at 3MPa for dopamine (DA), glutamate (Glu) and citrulline (Cit-NO) levels. DA and Glu levels refer to previous studies [4-6]. We considered citrulline as a good index of nitric oxide (NO) levels in the brain. The effect of nitrogen at 3MPa comparing basal values in atmospheric ambiance is noted in bold letters. The comparison between the basal effect of nitrogen at 3MPa and its effect combined with a chemical stimulation by retrodialysis is noted in italic letters.

Our observations reflected the balance between the uptake and release processes of arginine and citrulline. Therefore this should not be forgotten to appreciate NO synthesis. L-citrulline is presently well accepted as a nitric oxide index [32,40-43] as its production in the brain is directly linked to the NO production. But the interpretation of the L-arginine extracellular concentration remains discussed [24,27,43-47]. The arginine is not exclusive of NO synthesis and citrulline, and it is also used at the same time for other metabolic reactions [32]. Nonetheless, under nitrogen at 3MPa, there is less citrulline available to be recycled into arginine via the NO-citrulline cycle [29]. Hence, our discussion preferentially hinges on citrulline results, even if arginine remains essential to the understanding of NO development under nitrogen narcosis.

### Effect of nitrogen at 3MPa on striatal citrulline and arginine levels

Helium control was used to dissociate the effect of pressure from that of narcosis. Compared with nitrogen at 3MPa, helium at 3MPa does not induce narcosis, as it has very low narcotic potency [1]. Under helium, we observed a transitory increase-which disappeared at 3MPa-in citrulline and arginine levels that may be attributed to the variation of pressure. This short increase was not displayed under nitrogen during the compression phase what show the opposing effect of the narcotic gas action and the pressure per se effect [4,48,49]. Hence the lowered extracellular concentrations of citrulline and arginine, recorded under nitrogen, can be directly attributed to the effect of the narcotic potency of the gas.

While gathering data recordings in nitrogen narcosis, we firstly remarked that there is less glutamate for the potent induction of NO and citrulline production from arginine. Our previous paper highlighted that the NMDA-Receptor was not directly affected by nitrogen under pressure and the NMDA-activation should be able to activate NOS production linked to NO/citrulline synthesis. Nonetheless, we measured that striatal citrulline levels were not significantly increased by NMDA retrodialysis in nitrogen although it was in atmospheric conditions; neither were the arginine levels increased. Moreover, the arginine levels have shown no significant difference between data recordings in nitrogen, whether NMDA retrodialysis was used or not. We should conclude that the NMDA-evoked citrulline and arginine increases were counteracted by nitrogen narcosis once again, and that NO was not synthesized despite the NMDA-Receptor stimulation. Although it was not significant, graphics clearly show an increase in arginine and citrulline levels at the beginning of the NMDA stimulation that was counteracted when nitrogen raised 3MPa and its strongest narcotic effect. We should conclude that the nitrogen effect acts at a level affecting NO synthesis.

Nevertheless, taking into consideration both PFC-stimulation experiments conducted under pressurized nitrogen or not, extracellular citrulline and arginine levels remained unchanged. The striatal citrulline level remains raised and the striatal arginine levels remains reduced, while the glutamate levels were strongly reduced. This last comment led to reconsideration of the previous conclusion indicating that the citrulline increase can be counteracted by pressurized nitrogen. That is to say, NO/citrulline synthesis is possible in nitrogen at 3MPa and the mechanism involved in the citrulline decrease recorded in nitrogen narcosis is not directly affected by the gas. There could be a factor which is able to activate NOS activity while the PFC was stimulated but is not involved when using striatum-NMDA stimulation.

### Effect on neurotransmitter release

Briefly, in conventional conditions but not in nitrogen narcosis, the strong NMDA-R stimulation promotes dopamine, glutamate and citrulline/NO increases, due to the fact that NO synthesis is under glutamate control and dopamine regulation. As a result of these reduced levels, NO cannot enhance neurotransmitter exocytosis, and more particularly glutamate exocytosis at the presynaptic level. It could lead to a weak activity of thalamostriate and corticostriate pathways involving those nitrogen narcosis symptoms.

In other words, an increase in NO level, that is NMDA-dependent, could partly manage a decrease in synaptic vesicle endocytosis and later stages of recycling [13,25]. Hence, NMDA-evoked NO production could partly increase extracellular dopamine and glutamate levels. This status seems to be in accordance with the NMDA experiments. PFC stimulation results were also in accordance with such a hypothesis: in nitrogen exposure there was no more glutamate available for a NMDA-induced NO release, itself inhibiting DA re-uptake, but there was no dopamine available for recycling or being broken down into DOPAC (dihydroxyphenylacetic acid) and HVA (homovanillic acid).

Arginine availability, which is dependent on the activation of ionotropic non-NMDA receptors, controls the NMDA-induced nitric oxide synthesis: the arginine transfer from glial cells takes place on activation of glial ionotropic non-NMDA receptors and the predominant glial localization of arginine [27]. In narcosis, glutamate is not available for stimulating non-NMDA-Receptors and this lack could limit the arginine shuttle.

### Effect on basal ganglia pathways

This study was conducted in the striatum in charge of motor and cognitive function. It is the primary input nucleus of the basal ganglia which impinge on the whole basal ganglia pathways. We now highlight rat hypoactivity under nitrogen narcosis is concomitant to low levels of main striatal neurotransmitters (Table 3). The motor and locomotor disruptions recorded under nitrogen at 3MPa are linked to dopamine glutamate and a potent NO decreases, in regards of citrulline and arginine levels. The low glutamate release of corticostriate and thalamostriate projections, the principal glutamatergic striatal afferents, could now be linked to a sensitization of the NMDA receptor [18-20] in relation with the NO decrease. This could represent another retrograde regulation of postsynaptic zone [13] in the striatum.

## Conclusion

The current study was undertaken to examine the impact of nitrogen under pressure on the NO pathway, and to focus on glutamate and dopamine reduction in narcosis. We demonstrated that a reduction of striatal glutamate and dopamine levels recorded in nitrogen narcosis was coupled to a decrease in levels of arginine, the NO precursor, and of citrulline, the NO co-product. We suspected an NO decrease under nitrogen narcosis. Therefore, in narcosis glutamate is not available for stimulating either the non-NMDA or NMDA-receptors. Hence there is no glutamate-induced NMDA-R dependent NO synthesis and NO cannot enhance the strength of the glutamate signal spreading across basal ganglia pathways. Based on this evidence, we also conclude that the arginine shuttle between glial cells and neurons should not be able to supply arginine for citrulline/NO production. Nevertheless, we highlighted the glutamate-independent possibility of a citrulline/NO synthesis, but without identifying the missing key.

## Competing interests

The authors declare that they have no competing interests.

## Authors' contributions

All authors read and approved the final manuscript. All authors, NV, JEB and JJR participated equally to the study; in its design, its experimentations and its redaction.
